# Measurements and determinants of children’s exposure to background gamma radiation in Switzerland

**DOI:** 10.1093/jrr/rrac006

**Published:** 2022-03-28

**Authors:** Christophe L Folly, Antonella Mazzei-Abba, Astrid Coste, Christian Kreis, Ben D Spycher

**Affiliations:** Institute of Social and Preventive Medicine (ISPM), University of Bern, 3012 Bern, Switzerland; Graduate School for Health Sciences (GHS), University of Bern, 3012 Bern, Switzerland; Institute of Social and Preventive Medicine (ISPM), University of Bern, 3012 Bern, Switzerland; Graduate School for Health Sciences (GHS), University of Bern, 3012 Bern, Switzerland; Institute of Social and Preventive Medicine (ISPM), University of Bern, 3012 Bern, Switzerland; INSERM UMR 1296, Radiation : Defense, Health,Environment, Centre Léon Bérard, Bâtiment Cheney A 1er étage 28 rue Laennec, 69008 Lyon, France; Institute of Social and Preventive Medicine (ISPM), University of Bern, 3012 Bern, Switzerland; Institute of Social and Preventive Medicine (ISPM), University of Bern, 3012 Bern, Switzerland

**Keywords:** natural background radiation, terrestrial radiation, dosimetry, exposure measurements, low dose ionizing radiation

## Abstract

Epidemiological studies of children’s cancer risks associated with background gamma radiation exposure have used geographic exposure models to estimate exposure at their locations of residence. We measured personal exposure to background gamma radiation, and we investigated the extent to which it was associated with children’s whereabouts. We collected data on whereabouts and exposure to background gamma radiation over a 5-day period among children aged 4–15 years in Switzerland. We used D-Shuttle dosimeters to measure children’s exposure, and we asked parents to write their children’s activities in diaries. We used Poisson mixed-effects and linear regression models to investigate the association of hourly and overall doses, respectively, with children’s reported whereabouts. During the observed time, 149 participating children spent 66% indoors at home; 19% indoors away from home; and 15% outdoors. The mean personal exposure was 85.7 nSv/h (range 52.3 nSv/h–145 nSv/h). Exposure was 1.077 (95% CI 1.067, 1.087) times higher indoors than outdoors and varied by building material and (predicted) outdoor dose rates. Our study provides detailed information about children’s patterns of exposure to background gamma radiation in Switzerland. Dwelling building materials and outdoor dose rates are important determinants of children’s exposure. Future epidemiological studies may benefit from including information about building materials.

## INTRODUCTION

Several epidemiological studies have used geographic exposure models to estimate children’s exposures to background radiation and investigate cancer risks associated with low-dose radiation exposure during childhood [1–5]. Because the expected increases in cancer risks are small, large study populations are needed to detect them [6]. The use of model-based exposure estimates bypasses practical complications of measuring personal exposure among such large populations. Also, direct exposure measurements are burdensome for participants; they can reduce willingness to participate, potentially leading to biased study results [7]. Exposure models for background gamma radiation primarily attempt to capture the geographic variability of dose rates; these are commonly based on data from nationwide measurement campaigns. In contrast to models of exposure to domestic radon, many of which included building characteristics as predictors [8–11], building characteristics have rarely [5, 12] been used in modelling exposure to background gamma radiation. The extent to which geographic variation of background dose rates alone explain variations in personal exposures is unclear. Exposure assessment has been identified as the main source of uncertainty in the aforementioned studies of childhood cancer risks related to background radiation [7].

Likely factors that influence an individual’s dose of background gamma radiation—not determined by geology—include residential building construction materials, room geometry within the building and time spent indoors and outdoors [12–16]. Several studies measured the concentration of radioactive isotopes in building materials [17–22], yet their influence on doses received by inhabitants has been rarely assessed by dosimetric measurement. Although it seems unrealistic to incorporate building details, such as room geometries, into population-wide exposure models, certain available data, such as building type, could help refine estimates [23].

Indoor gamma dose rates are often reported to be higher than outdoor rates; however, the ratio appears to be context-specific. In Switzerland, two studies reported similar ratios of indoor-outdoor rates of about 1.17 [24, 25]. However, both studies were based on measurements from the area of Fribourg and might not be representative for the whole country. Moreover, neither of them directly measured exposure of individuals.

We conducted exposure measurements among children to: (i) quantify levels and variation of received doses, (ii) assess the extent to which instantaneous dose rates are affected by children’s whereabouts (including being indoors or outdoors) and building materials, and (iii) investigate the contribution of these factors to the overall doses received during the measurement period.

## MATERIALS AND METHODS

### Data

We collected data on the whereabouts and the exposure to gamma radiation of children aged 4–15 years. The included children are a nested sample within a nationwide survey, targeting a representative sample of 8331 children aged 0–15 years living in Switzerland. This larger sample was obtained by simple random sampling from the sampling frame of the Swiss Federal Statistical Office (FSO). The parents of 2841 children responded to the questionnaire; 891 respondents declared interest in participating in personal exposure measurements.

We contacted the parents of potential participants by phone in a random sequence. No two participating children were from the same family (or lived in the same household). During the phone calls, we explained the measurement procedure and fixed the time periods for the measurements. The necessary material for the measurements were sent to participating families by post. Children were instructed to wear two measurement devices—a UV dosimeter and a gamma ray-dosimeter—for five consecutive days. The consecutive days always began on a Wednesday to capture school day and weekend activities. Children wore the dosimeters on their chests using a strap tailored specifically for the study. We circulated 20 pairs of dosimeters among participating children from April to November 2019. We ceased contacting households in November because the value of UV measurements greatly diminishes during winter months. Background rates are known to be higher in the Swiss canton of Ticino [26]; households from canton Ticino were contacted at a slightly higher rate, thus oversampled. Ticino (South-East) was most affected by contamination in the Chernobyl accident aftermath; it has higher natural dose rates from terrestrial radiation—probably related to local geology—than the rest of Switzerland [27].

We used D-Shuttle dosimeters; these were designed to measure residents’ personal doses of gamma radiation after the Fukushima accident [28–30]. The dosimeters were manufactured by Chiyoda Technol Corp., Tokyo, Japan; they use Si-diode detectors to measure hourly personal dose equivalent H_p_(10) and total dose. In contrast to many dosimeters that record cumulative doses, D-Shuttle dosimeters also record interval doses at hourly intervals. The device records one count for about every 10 nSv of exposure. At a constant dose rate, the recorded counts can be assumed to follow a Poisson distribution. The relative uncertainty of hourly records at a dose rate of 100 nSv/h is thus about 30%; and for the cumulative dose over 5 days, about 3%. The D-Shuttle dosimeter properties (described in [31, 32]) are deemed suitable to measure levels of background radiation when aggregating several hours of measurements [32].

Parents were instructed to record their children’s activities and whereabouts during the measurement period in an activity diary that was sent to them together with the dosimeters. Each activity diary entry included an open-ended description, an address for the activity location and an indication whether the children were indoors or outdoors during the activity. We categorized entries into different types of location. We flagged them to indicate whether children were at home, at school, or visiting relatives or friends. We further flagged reported locations by category, such as mountain, forest, near a body of water, playground, other location, or missing information and we used a separate flag to indicate whether a child was transferring between two places (e.g. commuting to school). We geocoded reported addresses for all activity diary entries and computed distances to residential addresses. We checked and manually corrected geocodes retrieved from Google after comparison with the geocodes from two other services (OpenStreetMap and Swisstopo) and by comparing original with reverse geocoded addresses. The three mapping services retrieved geocodes within 500 meters of each other for 57% of all addresses. We manually corrected geocodes for about 20% of addresses. For the remaining addresses, either one of the three services failed to retrieve geocodes while the other two services were consistent or address information was insufficient to retrieve precise geocodes.

### Data cleaning

We removed unreasonably high D-Shuttle measurements. Although such outliers are rare, they can happen; for instance. For instance, due to external shocks to the dosimeter [28]. Any remaining values that were several times higher than expected dose rates were explored after linkage with activity diary entries.

We linked hourly D-Shuttle data with activity diary entries, which were of variable duration, by calculating weighted averages of the measured doses for each entry. The weights were defined by: (i) the duration of the overlap between the one hour interval of the D-Shuttle reading with the activity diary entry, and (ii) divided by the total duration of the activity diary entry. Since all participants responded to the questionnaires, we further used unique participant identifiers to link data with information collected through the questionnaires. These data included self-reported building characteristics, such as building type (e.g. apartment or detached house) and wall construction materials used for the child’s dwelling (categorized into brick only; brick and concrete; concrete only; wood only; and other).

### Estimation of outdoor dose rates

Our analyses included model-based estimates of outdoor dose rates as a covariate. These estimates were obtained for participants’ reported addresses using an exposure model adopted from a recent study about risks of childhood cancers related to external background radiation [33]. Doses for locations with insufficient address information for geocoding were imputed using the predicted doses at the residential address. The exposure model is based on a map of terrestrial radiation [34], a deterministic height-dependent function [35] to predict cosmic radiation and a map of the caesium contamination [36] that is corrected for the decay of caesium over time. For this correction, we applied an exponential decay rate that is calibrated to time-series spectrometry measurements taken at fixed locations [26] using a random effects log-linear model [33].

### Statistical analysis

We used mixed-effects Poisson regression models to investigate associations of hourly measurements with predicted outdoor dose rates for children’s whereabouts. We treated the dosimetric measurements as count data assuming that 10 nSv correspond to one count [32]. We fitted a sequence of models by incrementally refining categories of indoor and outdoor locations.

First in sequence we fitted a baseline model (model A0), including an overall fixed intercept, a random intercept varying by participant, and the estimated outdoor dose rates. We extended this model with an indicator for being indoors (model A1). Let ${Y}_{ij}$ be the rounded number of counts for participant $i$ during an activity $j$. We write model A1 as

$$ \mathit{\log}\left(E\left[{Y}_{ij}\right]\right)=\mathit{\log}\left({t}_{ij}\right)+{\beta}_0+{\gamma}_{0i}+{\beta}_1{X}_{ij}^{Estimate}+{\beta}_2{X}_{ij}^{Indoor} $$

where ${t}_{ij}$ is the duration of activity $ij$ (in hours), which here represents an offset, ${\beta}_0$ and ${\gamma}_{0i}$ are the fixed and random intercepts, respectively; and ${X}_{ij}^{Indoor}$ is an indicator variable taking on the value 1 and 0 for an indoor and outdoor activity, respectively. The exponential of ${\beta}_1$, $\mathit{\exp}({\beta}_1)$, can be interpreted as the ratio between outdoor and indoor exposures.

We refined model A1 by distinguishing indoor activities at home and elsewhere (model A2):

$$ \mathit{\log}\left(E\left[{Y}_{ij}\right]\right)=\mathit{\log}\left({t}_{ij}\right)+{\beta}_0+{\gamma}_{0i}+{\beta}_1{X}_{ij}^{Estimate}+{\beta}_1{X}_{ij}^{elsewhere}+{\beta}_3{X}_{ij}^{home} $$

where ${X}^{indoors}$ is split into indicators for being indoors at home (${X}^{home}$) and indoors elsewhere (${X}^{elsewhere}$). In the same manner, we then further distinguished measurements made indoors at school from those made indoors elsewhere (model A3). Similarly, we then further split ${X}^{home}$ to distinguish between dwelling types (model A4) first, then by building material (model A5) second. Finally, we included separate indicators for measurements done in forests, near bodies of water and in mountainous areas to distinguish them from other outdoor measurements (model A6), which in this model are captured by the intercepts. We selected the best fitting model according the smallest Akaike Information Criterion (AIC) and Bayesian Information Criterion (BIC). All Poisson mixed-effects models were fitted using the R package lme4 [37].

We also used linear regression models to assess the influence of the same variable subsets included in the sequence of models outlined above on the average dose rate measured by individual participants during the 5-day measurement period (models B0–B6). We used the adjusted-${R}^2$ to assess the proportion of variation in average dose rates received by individuals that is explained by the included variables. For this analysis, the indicator variables above were transformed to reflect the proportion of time spent at a given location category. For example, if a child spent 72 of the total 120 hours indoors at home, ${X}^{home}$ (in models B2, B3) would be 0.60, while ${X}^{wood}$ (in models B5, B6) indicating wood as building material would be 0.60 if the reported building material was indeed wood and 0.00 if another building material was reported.

We weighted models (B0-B6) by the language of the participants (as a proxy for region) to adjust for the overrepresentation of children living in the Italian-speaking canton of Ticino. The weights were calculated by dividing the number of potential participants in the original target sample obtained by the FSO by the number of actual participants.

## RESULTS

Of the 156 participants we mailed the measurement material, 149 conducted measurements and filled out the activity diaries. The total duration of recorded activities by the participants ranged from 32 to 141 hours; 122 participants conducted measurements for a at least 115 hours. All measurements were conducted between April and October 2019. Participating children lived predominantly in urban or semi-urban areas and slightly more than half were male (Table 1). Compared to the general population (as represented by the random sample received from the FSO), Italian-speaking participants were overrepresented due to a higher recruitment rate in the canton of Ticino; the oldest age group was underrepresented (data not shown). Furthermore, the lowest quintile of socio-economic position (area-based index derived for Switzerland [38]) was underrepresented (12%).

**Table 1:** Population characteristics of children participating in the study and summary statistics of the average exposures measured across population categories

				Measured Doses		
	Overall(%)	Duration(%)^+^	%Outdoors	Mean^*^	StdDev^*^	Range^*^
n	149(100)	17′133(100)	15.0	85.4	17.6	(52.3, 146.4)
sex						
male	81(54.4)	9′379(54.7)	15.5	88.5	19.6	(52.3, 146.4)
female	68(45.6)	7′754(45.3)	14.1	81.5	13.9	(55.4, 119.0)
age						
4–7	64(43.0)	7′247(42.3)	15.0	87.3	18.7	(57.1, 146.4)
8–11	59(39.6)	6′883(40.2)	15.5	84.7	17.7	(52.1, 132.3)
12–16	26(17.4)	3′004(17.5)	12.9	82.1	13.2	(57.5, 116.6)
language						
German	86(57.7)	9′900(57.8)	15.5	82.4	14.7	(55.4, 146.4)
French	43(28.9)	4′975(29.0)	14.2	78.4	11.9	(52.3, 113.1)
Italian	20(13.4)	2′258(13.2)	13.2	113.8	11.7	(94.0, 132.5)
Urbanization^a^						
(semi-)urban	115(77.2)	13′302(77.6)	14.8	86.7	18.5	(55.4, 146.4)
rural	34(22.8)	3′831(22.4)	15.0	80.8	12.9	(52.3, 116.6)
ssep_q^b^						
1	39(26.2)	4′533(26.5)	13.7	83.0	19.8	(55.4, 132.5)
2	29(19.5)	3′318(19.4)	14.4	86.6	17.7	(52.3, 131.2)
3	31(20.8)	3′498(20.4)	13.6	89.4	19.3	(61.7, 146.4)
4	32(21.5)	3′735(21.8)	16.9	82.8	13.8	(57.1, 132.3)
5	18(12.1)	2′049(12.0)	16.5	86.5	13.3	(66.5, 116.8)

a Urbanization of residential municipalities based on classification of the Federal Statistical Office [40]

b ssep_q; Quintiles of area-based socio-economic position [38]

+ Total time of available measurements in hours.

^*^Values in nSv/h

**Table 2:** Available measurements and summary statistics on measured dose rates by type of location

Location Type	N entries(%)	Duration(%)^+^	%Outdoors^++^	Mean^*^	StdDev^*^
Home	7076(54.3)	11 978(69.9)	5.5	85.7	13.3
School	1254(9.6)	1304(7.6)	14.0	79.9	22.1
Traveling	2707(20.8)	1073(6.3)	51.7	83.2	20.9
Relatives	495(3.8)	831(4.9)	21.8	84.6	17.3
Playground	165(1.3)	267(1.6)	83.6	82.5	15.4
Water	156(1.2)	238(1.4)	91.8	81.0	21.4
Mountain	123(0.9)	210(1.2)	49.5	93.8	16.2
Forest	47(0.4)	85(0.5)	100	78.6	8.2
Other	1117(8.6)	1317(7.7)	38.5	91.8	24.7
None^a^	56(0.4)	83(0.5)	60.6	78.0	10.6
Total	13,032^b^	17,133^b^	15.0	85.4	31.7

+ Total time of available measurements in hours.

++ % of time spend outdoors as reported by the parents. We indicated that time spent in a closed vehicle should not be classified as outdoors.

^*^Values in nSv/h

a No information regarding type of location reported in the activity diary.

b The separate rows sum up to more than the total because we assigned more than one type of location to some of the entries.

In total, 17 133 hours of measurements distributed over 13 032 activity diary entries were available after linking measurements to the activity diaries. We removed one entry due to an unreasonably high measurement reading about 100 times higher than the mean background (above 9000 nSv/h). Of all entries, 3304 (25.4%) referred to activities outdoors. The fraction of time spent outdoors was 15% (total measurement duration: 2572 hours), and it was similar among age categories, with the lowest value for the oldest children: 12.9% among children aged 12–16 vs 15.5% among children aged 8–11 and 15% among children aged 4–7. The most frequently reported location was home, which accounted for 7076 entries (54.3%) and 11 978 hours of measurements (69.9%), followed by school (entries: 9.6%; time: 7.6%) and travel-related activities (entries: 20.8%; time: 6.3%), which frequently referred to school commutes (Table 2).

**Table 3:** Wall building materials for the homes of participating families by dwelling type

Building material	Apartment (%)	Detached house (%)	Unknown(%)
Brick	5(9)	23(25)	0(0)
Concrete/Brick	13(24)	13(14)	0(0)
Concrete	9(17)	11(12)	0(0)
Wood	5(9)	11(12)	0(0)
Other	22(40)	34(37)	1(33)
Unknown	0(0)	0(0)	2(66)
Total	54(100)	92(100)	3(100)

**Fig. 1:**
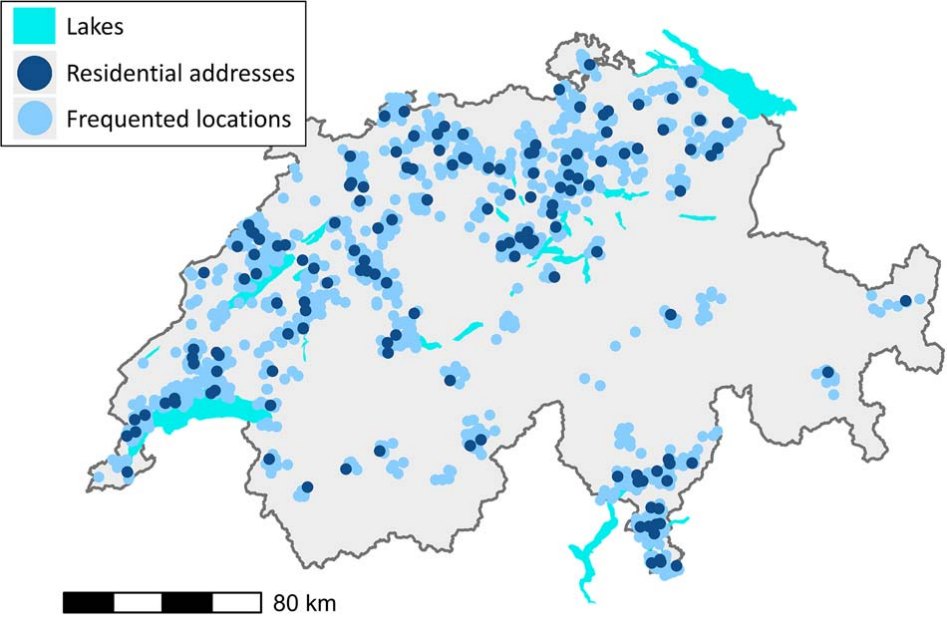
The residential addresses (dark blue) and frequented locations (light blue) of the 149 children who participated in the measurement campaign. All geocodes were jittered for anonymization by adding a random offset to the X and Y coordinates drawn from a uniform distribution between -3000 m and 3000 m. We excluded 36 foreign locations (in neighbouring countries) from the plot.

Considering entries that were successfully geocoded, about 64.6% of the time spent away from home (2887 of 4469 hours) was spent within 5 kms from home. Only around 15% of that time was spent more than 25 kms away from home. Figure 1 shows the residential locations of children and their frequented locations during the measurements. We were able to geocode the reported addresses for 11 699 (89.8%) activity diary entries, which corresponded to 16 447 (96%) hours of measurement. Geocodes could not be obtained for 1333 diary entries, which accounted for 686 hours of measurements. The most common reason for unobtainable geocodes was missing address information. Unobtainable geocodes accounted for 1231 (92.3% of missing geocodes) entries related to travel activities, which accounted for 561 hours (81.8%). Children’s dwelling types included detached houses (92; 62%); apartments (54; 36%); and unknown (3; 2%). Table 3 shows wall building materials by dwelling type.

**Fig. 2:**
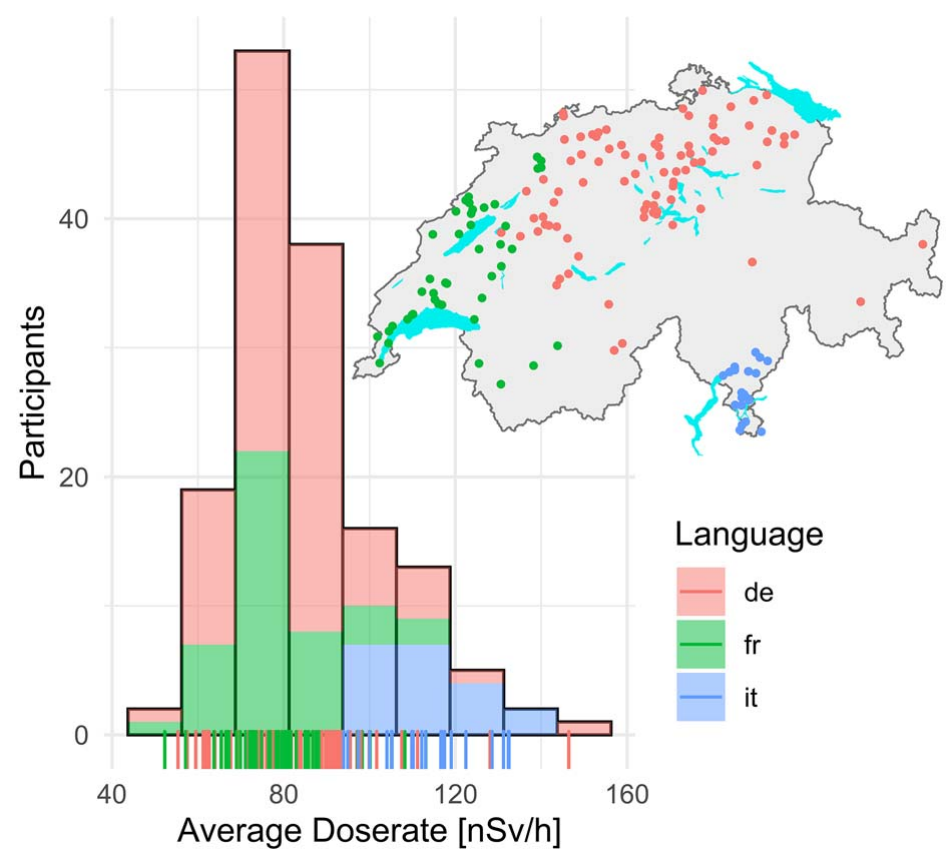
Histogram of average dose rates measured by the 149 participating children. Colours indicate the language (defined as the language used to communicate by local authorities) of the participants. The map indicates the residential address, jittered for anonymization by adding a random offset to the X and Y coordinates drawn from a uniform distribution between -3000 m and 3000 m. The lines with colour at the bottom indicate the individual average exposure over the measurement period.

Individual average dose rates over the measurement period ranged from 52.3 nSv/h–146.4 nSv/h, with a mean of 85.4 nSv/h and a median of 81.6 nSv/h (Table 1). Viewed as a population estimate, the mean dose rate has a standard error of 1.44 nSv/h and the 95% confidence interval (CI) is 82.6–88.2 nSv/h. Average doses rates tended to be higher in the Italian-speaking region compared to the French- and German-speaking regions; somewhat higher among males than females and in urban and semi-urban areas than in rural areas (Table 1). The distribution of personal mean exposure was right skewed with higher values observed mainly among children from the Italian-speaking canton Ticino (Fig. 2). The ${R}^2$ between mean dose rate measured over the whole period and mean dose rate measured at home was 0.919.

The mixed Poisson models that included additional information on the locations (indoor, type of building, etc.) were preferred by both the BIC and AIC (Table 4). Among these models, the most complex model (model A6), which included building materials of resident dwellings and outdoor measurements near bodies of water, in forests and in mountainous areas, was separately favoured by the AIC. The slightly less complex model (model A5) that considered building material but no specific outdoor locations was preferred by the BIC. Overall, the AIC and BIC decreased with increasing model complexity. These improvements were also reflected in a decreasing variance of the random intercept, which captures unexplained inter-individual exposure variation.

**Table 4:** Estimated coefficients and model comparison statistics of fitted Poisson-Mixed effects models, including the estimated dose rate as predictor

	Model A0	Model A1	Model A2	Model A3	Model A4	Model A5	Model A6
Fixed-Effects	Est.(SE)	Est.(SE)	Est.(SE)	Est.(SE)	Est.(SE)	Est.(SE)	Est.(SE)
intercept	1.893(0.022)	1.819(0.023)	1.804(0.023)	1.832(0.023)	1.811(0.024)	1.810(0.022)	1.805(0.023)
estimated dose rate^a^	0.261(0.016)	0.266(0.016)	0.280(0.016)	0.254(0.016)	0.275(0.016)	0.280(0.016)	0.293(0.017)
indoor(all, others)		0.074(0.005)	0.059(0.006)	0.081(0.007)	0.075(0.007)	0.087(0.007)	0.078(0.007)
indoor(school)				−0.007(0.009)	−0.009(0.009)	−0.016(0.009)	−0.024(0.010)
indoor(home)			0.077(0.005)	0.075(0.005)			
indoor(unknown)^b^^,^^c^					−0.150(0.021)	−0.150(0.031)	−0.157(0.031)
indoor(detached house)^b^					0.085(0.006)		
indoor(apartment)^b^					0.070(0.007)		
indoor(brick)^c^						0.151(0.009)	0.143(0.009)
indoor(concrete/brick)^c^						0.135(0.008)	0.129(0.009)
indoor(concrete)^c^						0.059(0.010)	0.052(0.011)
indoor(wood)^c^						−0.061(0.012)	−0.075(0.013)
indoor(others)^c^						0.051(0.006)	0.043(0.007)
outdoor(water)							−0.018(0.014)
outdoor(forest)							−0.025(0.024)
outdoor(mountain)							−0.073(0.019)
							
Random-effects							
Group	Variance	Variance	Variance	Variance	Variance	Variance	Variance
${\gamma}_{0i}$	0.0365	0.0365	0.0363	0.0366	0.0384	0.0301	0.0297
							
Model selection criteria							
AIC	108228.9	108000.1	107983.4	107891.4	107763.8	107533.4	107523.0
BIC	108251.3	108030.0	108020.8	107936.2	107823.6	107615.6	107627.7

ABR AIC, Akaike Information Criterion; BIC, Bayesian Information Criterion.

^*^Coefficients are presented on the log-scale. To transform back to predicted dose rates, use e.g. dose rate $=10\times \mathit{\exp}({\beta}_0+\beta 1+\dots )=10\times \exp (1.804+(0.280\times 1)+0.077)=86.8$ nSv/h for the average indoor dose rate at home based on model A2 and assuming an estimated outdoor dose rate of 100 nSv/h.

a Dose rate predicted based on the geocodes from the activity diary and the exposure model described in [33], Units 1 $=$ 100 nSv/h.

b Self-reported building type for the residence dwellings.

c Self-reported construction material used for the walls of the residence dwellings.

Measured exposures tended to be higher indoors than outdoors, with an indoor vs outdoor ratio of about 1.077 (95% CI 1.067–1.087) (model A1). There was evidence of difference between indoor and outdoor exposure only for home locations (indoor/outdoor ratio: 1.078, 95% CI:1.068–1.089), but not for school locations (0.993, 95% CI 0.975–1.011) (model A3). The indoor vs outdoor ratio in detached houses was slightly larger (1.089, 95% CI 1.077–1.101) than for apartments (1.072, 95% CI 1.058–1.087) (Model A4) (Table 5). The indoor vs outdoor ratios for buildings with walls constructed of brick (1.163, 95% CI 1.143–1.183) and a combination of brick and concrete (1.145, 95% CI 1.127–1.164) were higher than for buildings with walls made of concrete only (1.061, 95% CI 1.039–1.083) or of wood (0.941, 95% CI 0.919–0.964) (model A5).

**Table 5:** Measured indoor dose rates and modelled indoor/outdoor ratios by self-reported wall building material and by self-reported dwelling type

Number of dwellings(%)		^a^Mean indoor dose rate (SD)	Indoor/outdoor ratio(95%-CI)
Building material			
Brick only	28(19)	94.0(15.6)	^*^1.16(1.14, 1.18)
Brick/concrete	26(17)	98.0(20.4)	^*^1.14(1.13, 1.16)
Concrete only	20(13)	83.1(18.6)	^*^1.06(1.04, 1.08)
Wood only	16(11)	66.7(11.7)	^*^0.94(0.92, 0.96)
Other	57(38)	83.4(14.5)	^*^1.05(1.04, 1.06)
NA	2(1)	101.0(9.6)	^*^0.86(0.81, 0.91)
Dwelling type			
Apartment	54(36)	89.3(20.5)	^**^1.07(1.06, 1.09)
Detached House	92(62)	84.0(17.1)	^**^1.09(1.08, 1.10)
NA	3(2)	100.7(8.8)	^**^0.86(0.83, 0.90)
Total	149	86.2(18.6)	^***^1.08(1.07, 1.09)

a in nSv/h

^*^Based on model A5.

^**^Based on model A4.

^***^Based on model A3.

The (modelled) geographic variation of background gamma radiation was able to explain about 25% (adjusted-${R}^2$) of the variation in the average dose rates measured by the participants (Table 6). Adding time spent indoors without including building characteristic information did not increase the adjusted-${R}^2$ (models B1–B3). Including building type as predictor increased the adjusted-${R}^2$ to 28% (model B4). Further, including information about construction materials increased the proportion of variation explained by the model to 45% (model B5). The adjusted-${R}^2$ increased only slightly when adding separate terms for the time spent at specific outdoor locations (model B6). Geographic variation of outdoor dose rates remained the most important factor predicting exposure.

**Table 6:** Estimated coefficients and goodness of fit statistics of fitted linear models of individual average dose rate [nSv/h] over the measurement period

	Model B0	Model B1	Model B2	Model B3	Model B4	Model B5	Model B6
Fixed-Effects	Est.(SE)	Est.(SE)	Est.(SE)	Est.(SE)	Est.(SE)	Est.(SE)	Est.(SE)
intercept	21.5(8.7)	20.3(14.3)	20.2(14.3)	18.0(14.5)	19.8(14.1)	17.8(12.4)	4.5(14.5)
estimated avg. dose rate^a^	62.9(8.9)	62.9(8.9)	62.3(9.0)	61.6(9.0)	62.1(8.8)	66.8(7.8)	68.9(8.4)
%indoor(all, others)		1.5(13.8)	7.8(17.0)	15.8(18.6)	11.6(18.2)	5.00(15.9)	15.9(17.0)
%indoor(school)				−8.2(22.8)	−17.7(22.4)	0.9(19.6)	13.4(22.0)
%indoor(home)			0.9(13.9)	5.3(14.5)			
%indoor(unknown)^b^^,^^c^					31.1(20.0)	26.0(17.7)	38.2(18.9)
%indoor(detached house)^b^					0.1(14.3)		
%indoor(apartment)^b^					8.2(14.3)		
%indoor(brick)^c^						10.6(12.5)	22.4(14.5)
%indoor(concrete/brick)^c^						7.1(13.0)	19.3(15.0)
%indoor(concrete)^c^						−10.6(13.2)	0.9(14.9)
%indoor(wood)^c^						−22.6(13.0)	−10.6(14.8)
%indoor(others)^c^						−0.5(12.6)	11.3(14.4)
%outdoor(water)							27.0(40.2)
%outdoor(forest)							148.3(73.1)
%outdoor(mountain)							17.0(56.0)
							
Goodness of fit statistic							
adjusted-${R}^2$	0.251	0.246	0.243	0.243	0.280	0.451	0.458

^*^All models weighted by the language of the participants.

a Dose rate predicted based on the geocodes of the activity diary and the exposure model described in [33]. Units 1 $=$ 100 nSv/h.

b Self-reported building type for the residence dwelling.

c Self-reported construction material used for the walls of the residence dwelling.

## DISCUSSION

This study of personal exposure to background gamma radiation of 149 children revealed that both hourly doses and cumulative doses received over a period of several days are affected by children’s residential locations and their specific whereabouts. The preferred models included estimated outdoor dose rates, which (imperfectly) captured variation due to geographic location alone. Being indoors at home was associated with an average increase of 7.8% (95% CI 6.7–8.7%) in hourly doses compared to being outdoors. This increase was slightly higher in detached houses (8.9, 7.7–10.1%) than in apartments (7.2, 5.8–8.7%), and it was particularly large in homes with brick walls (16.3, 14.3–18.3%) as opposed to homes with wooden walls, which were associated with a decrease (−5.9, −8.1––3.6%) compared to outdoor exposure. We found no evidence of systematic indoor/outdoor differences at school locations. Although being indoors at home affected hourly rates, the proportion of time spent indoors at home did not contribute to explaining inter-individual differences in average dose rates over the measurement period, unless combined with building characteristics. Estimated outdoor dose rates combined with time spent inside dwellings of specified characteristics (building type and wall construction materials) explained about 45% of the variation in the average dose rates participants measured. On average, participating children spent 85% of their measurement period indoors and 70% at home (indoors and outdoors).

The overall mean dose rate of 85.7 nSv/h that was measured in this study corresponds to an annual average dose of 0.75 mSv. This is similar to the annual dose from terrestrial (0.35 mSv) and cosmic radiation (0.4 mSv) reported by the Swiss Federal Office of Public Health for the general population [26]. The distribution of the measured doses reflects the fact that a large proportion of the Swiss population lives in the Central Plateau which generally has lower levels of background radiation compared to the alpine region in the South, particularly the canton of Ticino. Estimates of outdoor dose rates based on a geographic exposure model were able to explain about 25% of the variation in the average dose rates measured by the participants of our study.

To our knowledge, no other study investigated the extent to which children’s measured exposure to background radiation is affected by their activity patterns in similar detail. One study used D-Shuttle dosimeters to investigate doses and exposure patterns of 216 high school students and teachers from Japan, France, Poland and Belarus [28]. The study focused on differences in exposure levels between countries and differences between school and home exposure. For the participating schools in Europe, the study found no evidence of differences between exposure in school buildings and at home. In our study, indoor home exposure was on average higher by about 8% than indoor school exposure.

We found a smaller ratio of indoor vs outdoor exposure than that previously reported for Switzerland [24, 25]. However, the values are consistent with a review of relevant studies [39] that reported values ranging from 0.6–2.3. All building materials provide some shielding against cosmic and terrestrial gamma rays. Indoor doses will be higher than outdoor if building materials contain enough radionuclides to outweigh this shielding. In our study, buildings with brick walls appeared to have higher indoor dose rates compared to buildings with concrete or wooden walls. This finding aligns with measurements of the radioactivity of building materials [17, 18, 21]. We thus speculate that the poor predictive value of dwelling type for estimating exposure in our study can be explained by similar usage of building materials for apartment buildings and detached houses in Switzerland.

Even though most of the measurements were conducted during summer months, the time spent outdoors in our sample was lower than what was assumed in older studies when estimating exposure of the general population (15% vs 20%) [24, 39]. Our study was suggestive of lower exposure at bodies of water and in forests, though the precision of these estimates was low. The direction of association, however, is plausible given that bodies of water act as a shield and that the organic material in forests conceivably has lower concentrations of radioactive material compared to other surroundings. Yet, the contribution of lower doses from time spent at such locations explaining differences in mean dose rates between participants was insignificant. The time spent outdoors at specific locations is likely too short to considerably affect cumulative doses over longer periods of time for most children.

A limitation of our study is that the information on factors that potentially modify exposure was largely self-reported, including information about children’s whereabouts and building characteristics of their home. This offered room for substantial misclassification. Our sample size was limited by the required effort and costs for conducting the measurements; the sampling cannot be considered random since participation involved two steps of parental consent. Our sample size was modest, yet large enough to estimate the overall mean dose rate with high precision (standard error 1.44 nSv/h). The dosimeters recorded a measurement every hour. Although such high time resolution is rarely used in studies of personal exposure, it did not allow us to align measured doses to individual activity diary entries. If a child spent half of an hourly measurement interval outdoors and the other half indoors, the recorded measurement would inevitably represent an average exposure of the two locations. Thus, the measurements are often weighted means of different exposure levels. This should not be a concern for activities lasting several hours. However, the contrasts between exposure levels are inevitably diluted if these change more frequently. The impact of this dilution on the study results may have been minimal though, given that more than 75% (50%) of the total measurement time was associated with activities lasting $\ge$1 hour ($\ge$3 hours).

The main strength of our study is the detailed information collected on the whereabouts of the children combined with a high temporal resolution of measurements. This allowed us to distinguish hourly exposure levels for different indoor and outdoor categories at finer granularity than has been previously investigated. The information on building materials turned out to be critical for explaining differences in hourly doses and average dose rates (and thus cumulative doses). Presumably, building materials are so influential because they affect doses incurred in the home, which by far outweigh all other contributions to overall doses. Also, the measurements were done over an extended period with both week and weekend days included. Aside from the intentional, overrepresentation of Italian-speaking participants, which we accounted for in the analyses, the sample provided a good representation of the population.

Our findings largely confirm the importance of geographic variation in outdoor gamma radiation levels and building characteristics for explaining the variability of exposure to external background radiation among the general population. Considering potential determinants, estimated outdoor dose rates and building materials of dwellings contributed most to improving model fit for hourly doses and average dose rates over the measurement period. In line with studies of radioactivity of building materials, brick walls were associated with the greatest increase of indoor doses. The actual concentrations of radionuclides in any building material may vary considerably depending on the procurement area. In our study, being indoors at school was not associated with higher exposure levels compared to outdoors. This may be due to a combination of greater shielding properties and/or lower concentration of radionuclides of construction materials and larger room volumes.

About one quarter of the variation in individual average dose rates was explained by the geographic variation in estimated outdoor dose rates alone. In this measurement study, we could estimate dose rates at the exact whereabouts of children throughout the day. In most epidemiological studies, however, only the residential address is available and misclassification due to children’s daily mobility patters is unavoidable. However, as children spend the bulk of their time in or near their homes, the degree of such misclassification is likely to be minor. Our finding confirms that over 90% of variation in personal exposure was accounted for by home exposure. Efforts improving exposure models should rather be directed towards a more accurate estimation of home exposure and indoor exposure. In our study, time at home is almost entirely spent indoors (94.5%). If possible, such efforts should attempt to include information about building characteristics. Building type was included in the exposure model for terrestrial radiation of a Finnish study with higher exposure levels assumed in apartments compared to detached houses [5]. In our study, the contribution of building type was minor. Rather, it appears that construction materials are the dominant factor and that the extent to which building type, which may be more readily available in some countries, captures information about building materials is context-specific, likely differing between countries.

Our study shows that the geographic variation of outdoor doses and construction materials of dwellings accounts for a considerable proportion of variability in children’s exposure to background gamma radiation in Switzerland. It is also likely to be the case in other countries where children spend the bulk of their time at home and indoors and the variety of building materials in use is comparable. For future epidemiological research, efforts should be made to increase the accuracy of exposure models for predicting indoor exposure at children’s dwellings, if possible, by including information on building materials.

## AVAILABLITY OF DATA AND MATERIALS

An anonymized data set containing the information used for these analyses can be made available to researchers upon reasonable request (ben.spycher@ispm.unibe.ch).
